# Blood-borne infections and pregnancies among women attending a sexual violence assistance center in Brazil: A 10-year retrospective study

**DOI:** 10.1371/journal.pone.0280419

**Published:** 2023-02-15

**Authors:** Chiara Musso Ribeiro de Oliveira Souza, Gustavo Ribeiro Lima, Angélica Espinosa Miranda

**Affiliations:** 1 Postgraduate Program in Infectious Diseases, Health Sciences Center, Federal University of Espírito Santo, Vitória, Espírito Santo, Brazil; 2 Department of Gynecology and Obstetrics, Health Sciences Center, Federal University of Espírito Santo, Brazil; 3 Department of Social Medicine, Health Sciences Center, Federal University of Espírito Santo, Brazil; Norwegian Refugee Council, JORDAN

## Abstract

**Introduction:**

Sexually transmitted infections (STI) and pregnancy can be consequences of sexual violence. In Brazil, around 50% of women victims of sexual violence do not undergo STI prophylaxis or emergency contraception.

**Objectives:**

To analyze socio-demographic and epidemiological profile, frequency of procedures performed, frequency of blood-borne infections (BBI), pregnancy, and legal abortion in women assisted by a sexual violence assistance center.

**Patients and methods:**

This 10-year retrospective cohort study (2010–2019) describes the socio-demographic and epidemiological profile and frequencies of clinical procedures, BBI, pregnancies, and legal abortions in 915 women assisted in a sexual violence assistance center in Brazil. We extracted data from the medical records and used descriptive statistics and chi-square and logistic regression.

**Results:**

A total of 93.3% (842/915) were residents in the Metropolitan Area of the capital, 80,83% (733/915) were brown-skinned or white, 42.4% (388/915) were adolescents (12–17 years old), 80.4% (736/915) were single, most had no children, average of 1.8 (±1.0 DP) children. About one-third (313/915) had not had previous sexual intercourse, 1.6% (10/653) were pregnant. Rape predominated with 92.0% (841/915), of which 51.5% (471/915) involved a known or related aggressor, mostly an acquaintance, followed by a stepfather or father. Recurrent cases were 24.0% (227/915).

**Clinical procedures:**

42.6% (390/915) were attended within 72 hours and received STI prophylaxis 43.4% (392/904); emergency contraception 38.6% (349/904); blood collection 71.6% (647/904). Prevalence: syphilis 0.3% (2/653); hepatitis B 0.2% (1/653); pregnancy 1.6% (10/653). Incidences: syphilis 1.1% (7/633); hepatitis B 0.8% (5/633); hepatitis C 0.6% (4/633); pregnancy 27.2% (172/633). There were no HIV cases. Trichomoniasis at 1.9% (2/108), HPV-induced cytological lesions at 4.7% (5/108), and bacterial vaginosis at 20.0% (21/108) were found on cervicovaginal samples. There were 129 legal abortions.

**Conclusions:**

The socio-demographic aspects and the characteristics of the aggressions in the studied population are like those described in the Brazilian national database, including the remarkable number of adolescents. STI prophylaxis and emergency contraception were performed in less than half of the women. The incidence of pregnancy was higher among those women reporting firearms threats and lower among those receiving STI prophylaxis. The frequency of legal abortion was higher than in national data. Public policies ensuring access to sexual and reproductive health rights and strategies to improve the quality of care for women victims of sexual violence and education improvement may decrease vulnerability to STI and unintended pregnancies.

## Introduction

Violence is considered by the World Health Organization (WHO) [1] to be one of the leading global public health problems. In addition, violence against women is one of the main forms of human rights violations [2]. Sexual violence is one of the categories of violence, defined by WHO as actions ranging from harassment to forced penetration, including different kinds of coercion and situations in which a person is unable to give consent [3]. It is important to mention that the definition covers, but is not limited to, violence within marriage or romantic relationships [3]. Sexual violence occurs worldwide [4], and since 2008, the United Nations has officially considered rape a war weapon [5]. WHO reports that in some countries, almost one in four women may experience violence perpetrated by an intimate partner and that almost a third of adolescent girls report that their first sexual experience has been forced [6]. The Centers for Disease Control and Prevention (CDC) reported that around 20% of women in the U.S. had been raped during their lives [7]. In Brazil, in 2019 there were 66.123 cases of rape and rape of vulnerable persons (one rape every 8 minutes), where 57.9% of victims were no older than 13 years old [8].

Sexual violence causes repercussions not only on physical health due to the risk of acquiring sexually transmitted infections (STI) but also on mental health [2, 6, 7]. The biopsychosocial consequences of sexual violence are difficult to measure as they affect most victims and their families and can produce intense and devastating, often irreparable, emotional effects [4]. Immediate medical assistance to people in sexual violence condition (ideally up to 72 hours after the assault) allows offering the prophylaxis of non-viral STI (gonorrhea, chancroid, chlamydia, syphilis, and trichomoniasis), viral STI (hepatitis B and HIV) and emergency contraception (which can be done up to 5 days after the assault), thereby avoiding future damage to women’s health [9–11]. However, health national database figures for 2011 from the Notifiable Diseases Information System (SINAN) show that 46.1% of adolescents and 63.8% of adults in sexual violence condition have prophylaxis for STI and 35% of adolescents and 44% of adults performed emergency contraception [12]. This study showed rates of 3.5% of STI and 7.1% of pregnancies due to rape [12].

An American population-based study showed a higher risk of STI diagnosis and treatment in women who suffered sexual violence, regardless of the time after the event [13]. It is known that the risk of acquiring STI depends on several factors, such as the type of violence suffered (vaginal, anal, or oral penetration), the number of aggressors, the type of exposure (single, multiple, or chronic), the occurrence of genital trauma, the woman’s age and susceptibility, hymenal condition, the presence of STI, pregnancy length and the form of coercion used by the aggressor [11].

According to WHO, sexual violence has been neglected by research [6]. In Brazil, few quantitative studies have been conducted, probably due to the difficulty of obtaining consistent and minimally qualified data [14]. We analyzed the socio-demographic and epidemiological profile, the frequency of procedures performed, and the frequency of BBI, pregnancy, and legal abortion in women assisted by a sexual violence assistance center. We presume that the frequency of STI and pregnancy may reflect the quality of sexual and reproductive health care of women in sexual violence conditions.

## Patients and methods

This is a retrospective cohort study including adolescent and adult women aged 12 who were assisted by a sexual violence assistance center in Vitória, Espírito Santo State, between January 01, 2010, and December 31, 2019. We extracted data from the medical records and used descriptive and analytics statistics in the analysis.

The sexual violence assistance center is the Assistance for Sexual Violence Victims Program (PAVÍVIS), an extension program of the Department of Gynecology and Obstetrics of the Health Sciences Center, Federal University of Espírito Santo. The base of operations since 1998 is the Cassiano Antônio Moraes University Hospital (HUCAM). The program works with a multidisciplinary team comprising gynecologists and obstetricians, nurse, social worker, and psychologists. It ensures access to STI prophylactic medication (azithromycin, ceftriaxone, metronidazole, benzathine penicillin, tenofovir + lamivudine, dolutegravir), emergency contraception (levonorgestrel preceded by anti-emetic) and legal abortion. It is also checked HBV and HPV vaccination *status*. Pregnant women receive information about their right to abortion or adoption, or prenatal care, and, usually, they remain at the assistance center until delivery in the university hospital. They also receive psychological care.

**Fig 1:**
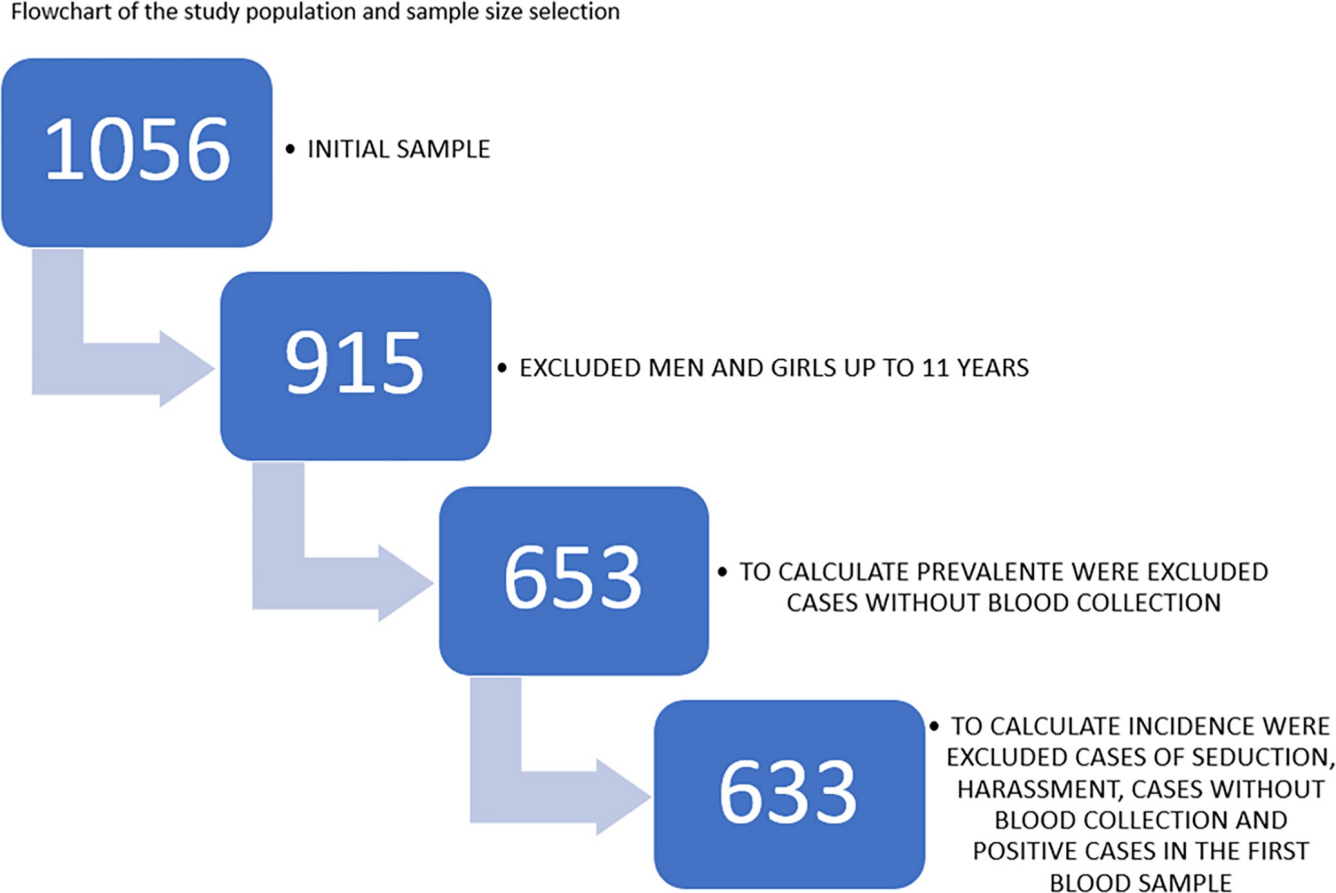
Flowchart of the study population and sample size selection.

The data were extracted from the notification sheets and medical records and entered into Excel spreadsheets. The variables are demographic information, clinical information, characteristics of aggression, procedures performed, and complementary laboratory tests. The software IBM SPSS Statistics version 24 was used to conduct statistical analyses. Data characterization was presented as frequency, percentage, minimum and maximum values, median, mean, and standard deviation. The frequencies of STI and pregnancy were calculated. To calculate prevalence, only women who underwent blood collection were included in the denominator, and only women with positive results on the first test were included in the numerator. To calculate incidence, only women who had a negative result for STI in the first blood collection were included in the denominator, and those with a positive test in the second examination were included in the numerator (Fig 1).

The second positive test was considered to be any positive test within six months of follow-up, excluding the first test. Fisher’s exact test was used for situations where expected values were below 5. The chi-squared test measures the association between independent categorical variables and outcomes. McNemar’s test associated with STI and the pregnancy test between the 1st and 2nd tests performed at first patient care and at the return for case follow-up.

For the study variables presenting a statistical significance of 10% in the chi-squared test, the unadjusted *odds ratio* was calculated and adjusted using the regression model. Multiple logistic regression with the Forward variable selection method was used to evaluate the association of outcomes with possible risk or protection factors. These associations were carried out considering the 1st and 2nd exams together and also the 2nd exam alone. The *alpha* significance level used in all analyses was 5%.

The use of secondary data and notification forms was approved by HUCAM management. The study was submitted and approved by HUCAM Research Ethics Committee under number 2.213.318/2017. We did not have contact with patients. Confidentiality of information was protected so that the patient’s identity was kept safe.

## Results and discussion

The study included 915 women in sexual violence conditions assisted by the PAVÍVIS program. The majority, 93.3% (853/915), were from Vitória Metropolitan Area towns. The socio-demographic characteristics of the women can be seen in Table 1.

**Table 1:** Socio-demographic characteristics of women in sexual violence situations assisted by PAVÍVIS between 2010 and 2019 (N = 915).

Characteristic	n	f %
**Age**		
12 to 17 years old	388	42.4
18 to 23 years old	144	23.0
24 to 29 years old	113	18.1
30 to 39 years old	90	14.4
40 or older	47	7.5
**Color**		
Brown	319	51.0
White	187	29.9
Black	93	14.9
Yellow	1	0.2
No data	26	4.2
**Marital Status**		
Single	493	78.8
Married / Consensual Union	83	13.3
Separated / Divorced / Widowed	44	7.0
No data	6	1.0
**Education**		
Primary school	104	16.6
Middle school	187	29.9
High school	237	37.9
College	60	9.6
Illiterate	6	1.0
No data	27	4.3

Regarding gynecological and obstetric history, the mean number of children of the women in the sample was 1.8 (SD ± 0.9), the number of spontaneous abortions was 1.5 (SD ± 1.2), and induced abortions 1 (SD ± 0). The mean age of coitarche was 15.9 (SD ± 3.8 years), the mean of sexual partners was 3.2 (SD ± 2.8), and 22.9% (210/915) were using contraception. About a third of the women (313/915) had not initiated sex life, and 1.6% (10/653) women were pregnant when they suffered sexual violence.

Around 25.0% (227/915) of the women in the study were victims of recurrent sexual violence. Only 2.6% (24/915) reported the use of illegal drugs, and 6.0% (55/915) related the use of alcohol. Regarding the characteristics of the assaults, 92.0% (842/915) of the cases of sexual violence in this sample were rape. Most episodes occurred in violent places, at in the metropolitan area. Sixty percent (550/915) were threatened by: hanging 0.3% (3/915), physical force or beating 4.5% (41/915), drug 5.0% (46/915), cutting/piercing 11.3% (103/915), fire gun 19.3% (177/915). Regarding the number of aggressors and whether they were known, 89.4% (818/915) reported one aggressor, in 51.5% (471/915) of cases the aggressor was an acquaintance or relative: 67.2% (307/471) acquaintance; 11.7% (55/471) stepfather; 7.0% (37/471) father; 4.9% (23/471) boyfriend; 3.4% (16/471) ex-husband or ex-boyfriend; 3.0% (14/471) institutional relationship; 2.1% (10/471) husband;1.5% (7/471) brother; 0.2% (1/471) son, and 0.2% (1/471) caregiver.

Table 2 shows the procedures performed in the women assisted by the program, 42.6% (390/915) were attended within 72 hours; 36.9% (338/915) received STI prophylaxis and emergency contraception.

**Table 2:** Procedures performed in women in situations of sexual violence assisted by PAVÍVIS between 2010 and 2019 (N = 904*).

Variables	Performed	Not performed	No available data
	n (%)	n (%)	n (%)
STI Prophylaxis	392 (43.36)	491 (54.31)	21 (2.32)
Emergency contraception	349 (38,60)	519 (57.41)	36 (3.98)
Cervical cytology	196 (21.68)	667 (73.78)	41 (4.54)
Vaginal discharge collection	10 (1.10)	827 (88.10)	67 (7.10)
Blood collection	653 (72.23)	189 (20.90)	62 (6.85)

*N excludes cases of seduction, harassment

Table 3 describes the frequencies of BBI and pregnancy in the population who underwent blood collection for analysis. It can be observed higher frequency of STI after the violence event.

**Table 3:** Frequencies of blood-borne infections and pregnancy in women in sexual violence situations assisted by PAVÍVIS between 2010 and 2019.

Blood-borne infection	Prevalence*	Incidence**
	n (%)	n (%)
Syphilis	2 (0.32)	7 (1.10)
Hepatitis B	1 (0.16)	5 (0.78)
Hepatitis C	0 (0.00	4 (0.63)
Pregnancy	10 (1.60)	172 (27.17)

* N = 653 (includes all cases of sexual violence and excludes cases where there was no blood collection)

** N = 633 (excludes cases of seduction, harassment, cases where there was no blood collection and positive cases in the first blood sample)

Trichomoniasis was identified in 1.9% (2/108) of women who underwent cervical cytology collection. Cervical cytology results presented the following lesions: 2.8% (3/108) LSIL and 1.9% (2/108) HSIL, both HPV-induced cytological lesions. About 20% (21/108) of the cytologies had predominant flora of coccobacilli, *G*. *vaginalis*, and *Mobiluncus*, microbiota that characterizes bacterial vaginosis. Regarding the women’s sexual history, those reporting more than one sexual partner had a 1.3 times increased chance (odds) of testing positive for syphilis on the 2nd test compared to those reporting only one sexual partner (p = 0.036). And those reporting the age of coitarche being 13 years or older had a significantly lower odds (OR 0.79, 95% CI 0.63–0.99) of testing positive on hepatitis B (2nd test) compared to those reporting age of coitarche being 12 years or younger (Table 4).

**Table 4:** Variables associated with blood-borne infections and pregnancy in women in sexual violence situations assisted by PAVÍVIS between 2010 and 2019 (N = 904**).

Dependent variables	Independent variables		P-value*	OR	95% CI for OR	
					Lower limit	Upper limit
Syphilis (any positive test)	Number of aggressors involved in violence	One	-	1	-	-
		Two	0.998	1	0.000	
		Three or more	**0.030**	14.751	1.296	167.930
Hepatitis B (any positive test)	Number of aggressors involved in violence	One	-	1	-	-
		Two	0,997	1	0.000	
		Three or more	**0.033**	12.329	1.218	124.808
Syphilis (2nd test (positive)	Number of sexual partners					
	One			1	-	-
	More than one		**0.036**	1.280	1.016	1.612
Hepatitis B (2nd test (positive)	Age of coitarche					
	< = 12			1	-	-
	13 or older		**0.039**	0.792	0.635	0.989
Pregnancy (2nd positive test)	Age group	< = 23 years old	**0.004**	7.698	1.886	31.424
		24 to 29 years old	**0.004**	6.926	1.846	25.985
		> = 30 years old		1	-	-
	Firearm	No	-	1	-	-
		Yes	**0.007**	5.675	1.616	19.936
	STIs prophylaxis	No	-	1	-	-
		Yes	**< 0.001**	0.008	0.002	0.032
HPV-induced cytological lesion	Number involved in violence	One	-	1	-	-
		Two	0.843	1.376	0.059	32.183
		Three or more	**0.043**	22.591	1.104	462.151
Bacterial vaginosis	STIs prophylaxis	No	-	1	-	-
		Yes	**0.025**	0.339	0.132	0.870

(*) Multiple logistic regression with *Forward* variable selection method; or—*Odds Ratio*; (1) reference category; significant if p<0.050. Variables included in the model—Town of residence, Town of occurrence, Age group, Race/color, Marital status, Formal education, Disabilities or disorders?, If so, what kind of disorder?, Pregnant, Number of children, Number of miscarriages, Age of coitarche, Number of sexual partners, Use of contraception, Use of drugs by the victim, Has it occurred other times?, Bodily force or assault, Piercing-cutting object, Medication, Firearm, STIs prophylaxis, Emergency contraception, Number involved in violence, Aggressor kinship, Drug use by aggressor. N excludes cases of seduction and sexual harassment and cases in which there was no blood collection.

(* *) N excludes cases of seduction and sexual harassment

Regarding the characteristics of the aggressions, the occurrence of three or more aggressors increased the chance of a diagnosis of syphilis by 14.7 times (p = 0.030) and of hepatitis B by 12.3 times (p = 0.033), in addition to increasing the chance occurrence of HPV-induced cervical cytological lesion by 22.6 times (p = 0.043), when compared to violence perpetrated by one aggressor. We found 27.5% (172/653) of pregnancies resulting from sexual violence, with a higher chance of pregnancy (7.7 times, p = 0.004) between ages < = 23 and between ages 24 and 29 (6.9 times, p = 0.004) when compared to those older than 29 years of age. Among these pregnant women, 5.8% (10/172) received emergency contraception and still became pregnant; 75.0% (129/172) underwent legal abortion. Among women who were threatened, the use of firearms was associated with 5.7 times (p = 0.007) higher chance of becoming pregnant (Table 4).

The socio-demographic data of the women included in the study are similar to the Brazilian data described in SINAN in 2011 and in the Brazilian Public Security Yearbooks of 2020 and 2021 [7, 15], in which the vast majority of women in sexual violence condition have low education, children, and, adolescents account for more than 70% of the victims, most are white or brown-skinned, and single women are more targeted than married women [8, 12]. About a third of the women (313/915) had not yet started their sex life when they suffered sexual violence, a similar figure to the one described by Rosa et al. [16] and by WHO [3], which can be related to sample characteristic, once we included girls from 12 years old, age at which adolescence begins according to the Statute of Children and Adolescents [17].

It is noteworthy that 42.4% of the sample were between 12 and 17 years old; we point out that 25.7% (235/915) were between 12 and 14 years old, an age that comprises the so-called rape of the vulnerable by Brazilian law (even if consent is given) [18]. In Brazil, in situations of violence against adolescents and children, the Child and Adolescent Guardianship Council must be notified, as recommended by the Child and Adolescent Statute [17], which triggers the health surveillance, and the victims are admitted at health services to receive prophylaxis and prenatal care. It can be a reason of the big number of adolescent girls in the sample. Nonetheless, data from the 2021 Brazilian Public Security Yearbook [15], which sources are police data, show that 60.6% of rape victims in Brazil in 2020 were only up to 13 years old. Unfortunately, early sexualization and sexual violence against children and adolescents is still an issue surrounded by taboos, fears, omissions, and even indifference in various segments of society [19]. On the other hand, SINAN data includes women victims who sought help from health services, so it is possible to occur an underrepresentation of adult women who have become invisible to society’s eyes because they have not sought help, given the taboos, fear, and shame involved [20].

Generally, 70% of sexual violence cases are committed by relatives, boyfriends, or friends/acquaintances of the victim; unknown offenders become the main perpetrators as the victims’ age increases [12]. WHO [1] recognizes multiple factors that interfere at individual, community, and social levels, pointing out risk factors for a man to commit sexual violence: being a member of gangs, use of alcohol or drugs, antisocial personality, parental violence, physical or sexual abuse in childhood, low educational level, gender imbalance. The so-called rape culture brings reflections on the social dynamics of gender relations [21]. It is, in general terms, the sharing of values, beliefs, and practices about gender roles and the sexual interactions that not only allow but also structure unequal relationships in which an active sexual interest must conquer and subdue the object of desire [21]. This culture, understood as a universe of shared practices and symbols that justifies or minimizes the seriousness and social dimension of rape and other forms of abuse, makes possible and naturalizes countless violations of girls and women [21]. Addressing this problem requires cooperation between the health, education and justice sectors, intervening in communities to change gender behaviors and improving women’s social and economic conditions [1, 20].

According to Cerqueira & Coelho [12], threat and bodily force/assault are strongly present and more frequent the higher the victim’s age group is, whether the aggressor is known or not. It is known that the use of blunt or cutting objects also increases the higher the age group is, and it is always higher when the aggressor is unknown [12]. The threat with a fire gun, which can inhibit the woman’s resistance to the perpetrator, was associated with 5.7 times (p = 0.007) higher chance of becoming pregnant (Table 5).

**Table 5:** Frequencies of performed clinical procedures and the incidence of blood-borne infections, pregnancies, and legal abortions, by period, in women in sexual violence situations assisted by PAVÍVIS between 2010 and 2019.

Variables	2010–2012	2013–2015	2016–2018	2019–2020
**Frequency of procedures performed in the period**				
Blood infections tests performed	146/198 (73.7%)	131/178 (73.6%)	240/289 (83.0%)	136/183 (74.3%)
STI prophylaxis administered	102/224 (45.5%)	110/238 (46.2%)	134/263 (51.0%)	46/159 (28.9%)
Cervical cytology collected	46/201 (22.9%)	37/212 (17.5%)	58/300 (19.3%)	55/150 (36.7%)
Vaginal discharge collection	2/191 (1.0%)	2/177 (1.1%)	3/286 (1.0%)	3/183 (1.6%)
Emergency contraception	88/219 (40.2%)	83/130 (63.8%)	112/298 (37.6%)	66/164 (40.2%)
**Incidence data of blood infections, pregnancy, and legal abortion**				
Blood infections	06/144 (4.2%)	05/130 (3.8%)	03/239 (1.3%)	02/136 (1.5%)
Pregnancy after the violence	29/172 (16.9%)	41/140 (29.3%)	59/251 (23.5%)	43/117 (36.8%)
Legal abortion	15/29 (51.7%)	31/41 (75.6%)	56/59 (94.9%)	27/43 (62.8%)

The objective of prophylaxis in sexual violence victims is to prevent and treat the most prevalent STI and pregnancy, and the Brazilian Ministry of Health recommends protocols and technical standards for this type of assistance which are followed by the PAVIVIS team [9–11]. Since 2001, the inclusion of violence–domestic, sexual, and other forms of violence–in the list of compulsorily notifiable diseases by the Brazilian Ministry of Health implemented the National Policy for the Reduction of Morbidity and Mortality from Accidents and Violence made data from health sector become visible, sometimes more than those from police sector [20]. In parallel, several laws have reinforced and guided health assistance to sexual violence victims throughout the country [22].

Since 2013, Brazilian law has ensured mandatory and immediate care in the Unified National Health System (UNHS) for people in sexual violence condition, and public hospitals must provide immediately: emergency contraception, diagnosis, and treatment of genital injuries, medical, psychological, and social support, prophylaxis of non-viral and viral STI, access to information about legal rights and health services available in the public health system [22]. There is a gap between the mandatory law and its implementation in the studied clinic, data are described on Table 5. The law was important to give visibility to this problem and providing a legal instrument to push for the clinic’s implementation, but despite the law, our clinic and many others in the country have difficulties to support the recommended implementation [4, 12]. In Brazil, as all health services are free of charge, public clinics and hospital are in charge to provide the procedures and several times, unfortunately, it does not happen adequately. Regardless these challenges, higher number of women have had access to legal abortion in our clinic in the last years, despite the covid-19 pandemic. It shows the law had some effect in the offered care, but there were still many women who did not have access to the procedures, showing we have a long way to go to improve our assistance.

Another finding in this study was the low percentage of patients who underwent prophylaxis procedures for bacterial and viral STI, around 40.0%. The vast majority had blood samples collected. Evidence collection, medico-legal examination, and forensic documentation were not performed at our hospital or ambulatorial settings, which explains only 1.1% of vaginal samples collected. These procedures occur at the medic-legal institute (at the police office). A woman who received STI prophylaxis had 99.2% (p<0.001) less chance of becoming pregnant and 66.1% (p = 0.025) less chance of developing bacterial vaginosis than one who did not receive prophylaxis. It is worrying that less than 50% of women in sexual violence receive STI prophylaxis. Most of the women in the sample were young, had low education, and knew the attacker, characteristics associated with a lower chance of receiving prophylaxis for STI [12]. Around 25% suffered recurrent sexual violence, which also decreases the chance of receiving prophylaxis for STI [12]. The low percentual of victims receiving STI prophylaxis is similar to those Brazilian data described by SINAN [12], which evidences a health system failure in women’s health care.

Initial screening for STI showed less than 1.0% of cases of syphilis and hepatitis B, and subsequent screenings showed incidence levels of 1.1% for syphilis, 0.8% for hepatitis B, and 0.6% for hepatitis C (Table 3). There was no testing for *Chlamydia trachomatis* and *Neisseria gonorrhoeae* during the study period due to budget constraints, and it was a limitation on the quality of health assistance. Testing for these microorganisms, available by UNHS in 2021, will improve the quality of health assistance in Brazil.

The risk of acquiring an STI depends on the type of penetration, the number of aggressors, how often the aggression occurs, and the woman’s age and susceptibility [2, 9]. For Drezett et al., [23] considering all the possible specific risks, approximately 50% of women victims of sexual violence face some STI. According to the Brazilian Ministry of Health [2], this rate varies from 16 to 58%; SINAN data [12] indicate 3.6% of STI cases are secondary to rape. Delziovo et al., [24] analyzing data from Santa Catarina State, Brazil, between 2008 and 2013, found 3.0%, 5.8%, and 2.4% of STI due to sexual violence, in the age groups 10 to 14, 15 to 19 and older than 20, respectively.

Less than 50% of women receiving STI prophylaxis do not seem to match the low frequency of STI found. Authors point out that there are data on the rate of STI acquisition after sexual violence; most data are on STI prevalence on initial evaluation [25–29]. However, sexual violence is often one of the biggest predictors of STI [13]. These authors showed that women who experienced sex without consent were more tested for STI and showed higher rates of herpes, genital warts, and chlamydia than the group of women who never had the same experience; that sex without consent increased the risk for diagnosis and treatment of STI, and that the population is susceptible, although STI cannot always be attributed to the event of sexual violence [13].

The frequency of STI in rape victims was also described in other studies. Hepatitis C: 1.4 to 3%, hepatitis B: 3%, gonorrhea: 3.5 to 12%, trichomoniasis: 3.1 to 22%, syphilis: 2.5%, 2suffer sexual crimes [28, 29]. In our study, a greater number of sexual partners increased the chance of contamination by syphilis and an increase in the age of coitarche decreased the chance of contamination by hepatitis B. Although HBV vaccination in Brazil, it’s important to point out that Espírito Santo State, where the study took place, presents high endemicity of HBV [30]. Regarding the characteristics of sexual violence, we found that the occurrence of three or more aggressors increased the chance of a diagnosis of syphilis by 14.7 times and of hepatitis B by 12.3 times, in addition to increasing the chance of presenting HPV-induced cervical cytological lesion by 22.6 times compared to violence by one aggressor. Brazilian data have previously shown an increase in STI cases when more than one aggressor was envolved [24] and a major frequency of more than one aggressor among adolescents [12]. It is remarkable that the occurrence of gang rape corresponded to 15.8% of all cases of SINAN data in 2014 [12, 20].

We did not find any prevalent or incident cases of HIV. HIV infection represents the main concern for about 70% of victims of sexual violence [31]. Well-conducted studies indicate that the possibility of HIV contamination ranges from 0.8 to 2.7%, comparable to the risk found in other forms of single sexual exposure or in occupational accidents among health professionals [9–11, 31]. In South Africa, was observed an increase from 0.9% to 6.4% in the percentages of positive tests for HIV among women in sexual violence condition, with more frequent cases among young people aged 16 to 20 [32]. It is estimated that 20 thousand young women and girls in Uganda will be infected with HIV each year as a result of rape [28]. One of the factors contributing to this forecast is the use of rape as a war weapon in Africa [5, 28, 32].

An increasing number of women seek health services for protective measures promptly, but an important percentage is not given adequate medication for STI prophylaxis [12, 24]. This inability to assist approximately half of the women in sexual violence conditions reveals a failure to ensure reproductive rights involving health policies, training of medical students and health professionals, and dissemination to the population of the importance and availability of early care provided by UNHS for people in sexual violence conditions [11].

Around 40% of the women in the study did not receive emergency contraception. Data from SINAN [12] show that 35 to 40% of women victims of sexual violence perform emergency contraception. Delziovo et al. [24] show that around 30% were not attended to in up to 72 hours and did not receive emergency contraception. A surprising 27.8% were attended to in up to 72 hours but did not receive emergency contraception. Emergency contraception, although a women’s right and one of the most important actions in immediate assistance after sexual violence, is ignored and not rarely omitted for unjustifiable reasons [33].

A Brazilian study in Santa Catarina State [24] showed 7.6% of pregnancies due to sexual violence, a higher rate in the group that was attended to 72 hours after the event and did not receive emergency contraception. We found 27.2% of pregnancies resulting from sexual violence, with a higher chance of pregnancy (7.7 times, p = 0.004) between ages 18 and 23 and between ages 24 and 29 (6.9 times, p = 0.004) (Table 5). We consider this percentage to be high, compared with data of Rosa et al. [15] (2.82%), SINAN, [12] (7.1%), Delziovo et al. [24] (3.8 to 10.8% depending on the age group), and the Brazilian Ministry of Health [2], which estimates pregnancy risk from rape to be between 0.5 and 5%. It is known that the risk of pregnancy resulting from sexual violence depends on the victim’s age, coincidence with her fertile period, whether the violence was an isolated or ongoing event and whether the victim was using contraceptive methods, knowing or not the aggressor [2]. Recurrence is more common among adolescent victims [12], which may have contributed to the high occurrence of pregnancies. We emphasize that health assistance for sexual violence victims must include both STI and emergency contraception to reflect good health care.

Another point regarding the lack of access to sexual health and adult women underreporting may be the position occupied by women in society, which is relevant when thinking about how victims cope with violence [34]. The context in which women suffer chronic violence, or the delay in reporting it or seeking health services, must be considered once they belong in a patriarchal Judeo-Christian culture that historically tends to blame and discredit women, especially when it comes to sexual issues [12]. It’s common to blame women who suffer violence as provocateurs/culprits of that situation, ascribing it to some behavior or attitude classified as inappropriate [12]. Fear of stigmatization can lead to delays in seeking medical care [12].

Of the 172 women who became pregnant after abuse, 129 (75%) interrupted pregnancy. Cerqueira & Coelho [12] show that 5% of adolescents and 19.3% of adults who suffered sexual violence had legal abortions, a percentage much lower than that shown in our sample. This may reflect the characteristics of the sexual violence assistance center, a benchmark for legal abortion in Espírito Santo State.

The limitations of this study have to do with the small sample size and the use of secondary data, which, due to the stigma, embarrassment to seek, and difficulty to access help, may have caused underreporting of adult women. But it is important to bring up the invisibility of these women in the health and police sectors. The program has many limitations, but it is a pioneer health center. It has assisted several women over the last 23 years, also allowing the spreading of skills in this kind of assistance. These data can highlight the program and help to improve the quality of its assistance. Low rates of STI can be explained by not testing for *C*. *trachomatis* and *N*. *gonorrhoeae*, this is undoubtedly a limitation of the study. Nonetheless, the dissemination of these data, bearing in mind their limitations, is important because it is worrying that many women do not access sexual and reproductive health rights, which integrates the list of fundamental guarantees recognizing sexual and reproductive rights as human rights [4, 11], once Brazil has an international commitment to promote public policies capable of ensuring reproductive rights (the right to sexual and reproductive health) [4].

## Conclusions

The socio-demographic aspects and the characteristics of the aggressions in the studied population are similar to those described in the national database. The number of adolescents in the sample is remarkable and brings reflections on early sexualization, rape culture, and adult women victims’ invisibility. STI prophylaxis and emergency contraception were performed by around 40% of the women. Both data on prophylaxis are similar to those described in the national database. The majority had blood samples collected, and a small percentage had vaginal samples collected. The prevalence and incidence of STI were low, and there was an association of STI with three or more aggressors committing violence, number of sexual partners, age of coitarche, and STI prophylaxis. The use of firearms increases the risk of pregnancy. STI prophylaxis, on the other hand, decreased the chance of pregnancy. The frequency of pregnancy resulting from sexual violence was surprisingly high. The frequency of legal abortion among these pregnancies was higher than reported in the national database. We hope that this study will draw attention to the need to implement public policies guaranteeing reproductive rights (sexual and reproductive health rights) and to develop strategies that will enable wide access and better quality of health care for women in sexual violence conditions, thereby reducing their vulnerability to STI and unwanted pregnancies.

## Supporting information

S1 Data (XLSX)
